# Protein Inhibitor of NOS1 Plays a Central Role in the Regulation of NOS1 Activity in Human Dilated Hearts

**DOI:** 10.1038/srep30902

**Published:** 2016-08-02

**Authors:** Esther Roselló-Lletí, Estefanía Tarazón, Ana Ortega, Carolina Gil-Cayuela, Ricardo Carnicer, Francisca Lago, Jose Ramón González-Juanatey, Manuel Portolés, Miguel Rivera

**Affiliations:** 1 Cardiocirculatory Unit, Health Research Institute Hospital La Fe (IIS La Fe), Valencia, Spain; 2 Department of Cardiovascular Medicine, University of Oxford, United Kingdom; 3 Cellular and Molecular Cardiology Research Unit, Department of Cardiology and Institute of Biomedical Research, University Clinical Hospital, Santiago de Compostela, Spain

## Abstract

An essential factor for the production of nitric oxide by nitric oxide synthase 1 (NOS1), major modulator of cardiac function, is the cofactor tetrahydrobiopterin (BH4). BH4 is regulated by GTP cyclohydrolase 1, the rate-limiting enzyme in BH4 biosynthesis which catalyses the formation of dihydroneopterin 3′triphosfate from GTP, producing BH4 after two further steps catalyzed by 6-pyruvoyltetrahydropterin synthase and sepiapterin reductase. However, there are other essential factors involved in the regulation of NOS1 activity, such as protein inhibitor of NOS1 (PIN), calmodulin, heat shock protein 90, and NOS interacting protein. All these molecules have never been analysed in human non-ischemic dilated hearts (DCM). In this study we demonstrated that the upregulation of cardiac NOS1 is not accompanied by increased NOS1 activity in DCM, partly due to the elevated PIN levels and not because of alterations in biopterin biosynthesis. Notably, the PIN concentration was significantly associated with impaired ventricular function, highlighting the importance of this NOS1 activity inhibitor in Ca^2+^ homeostasis. These results take a central role in the current list of targets for future studies focused on the complex cardiac dysfunction processes through more efficient harnessing of NOS1 signalling.

Heart failure (HF) is an increasingly prevalent clinical problem with high rate of morbidity and mortality in industrialised countries, and no curative treatment is currently available. Dilated cardiomyopathy (DCM) is one of the most frequent causes of HF. This severe pathology of unknown aetiology is characterised by dilation and systolic contractile dysfunction, with an increase in ventricular mass and volume and wall thickness^1^,^2^. The role of the free radical signalling molecule, nitric oxide (NO), in modulating cardiac function is well established^3^. This molecule is constitutively released in cardiomyocytes by both neuronal and endothelial isoforms of nitric oxide synthase (NOS1 and NOS3, respectively). NOS1, the major modulator of cardiac function and intracellular Ca^2+^ fluxes, has been extensively studied in experimental models^4^,^5^,^6^. An essential factor for the production of NO by NOS1 is the presence of the cofactor tetrahydrobiopterin (BH4). BH4 is regulated by GTP cyclohydrolase 1 (GCH1), the rate-limiting enzyme in BH4 biosynthesis which catalyses the formation of dihydroneopterin 3’triphosfate from GTP, producing BH4 after two further steps catalyzed by 6-pyruvoyltetrahydropterin synthase and sepiapterin reductase (SPR)^5^,^7^,^8^. However, there are other essential factors involved in the regulation of NOS1 activity, such as protein inhibitor of NOS1 (PIN: *DYNLL1* gene), calmodulin, heat shock protein 90, and NOS interacting protein^9^,^10^,^11^. Although we have studied this system in ischemic human hearts^12^, all these molecules has never been analysed in DCM.

Here, we focused on the assessment of NOS1 activity and cardiac tetrahydrobiopterins synthesis in left ventricular tissue samples obtained from DCM patients and compared the results with non-diseased controls (CNTs). We also identify differentially expressed genes closely involved in this process using the sensitive and powerful technique of RNA sequencing (RNA-seq).

## Results

### Clinical characteristics of patients

We analysed a total of 20 DCM human hearts obtained from patients undergoing cardiac transplantation, who had age of 48 ± 13 years and an NYHA functional classification of III–IV. Patients had previously been diagnosed with significant comorbidities, including hypertension (21%), and diabetes mellitus (18%). Table 1 summarises the clinical characteristics of the DCM patients. CNT samples were acquired from 10 non-diseased donor hearts. The CNT group mainly consisted of males (80%), with a mean age of 47 ± 16 years.

### Gene expression analysis

Differences in transcriptome levels between DCM and CNT samples were investigated through a large-scale screening of 23 heart samples (13 from DCM patients, 10 from CNTs) using RNA-seq technology. On comparing the two groups, we found 1628 differentially expressed genes, of which 596 were upregulated (≥1.5-fold increase; *P* < 0.05 for all) and 1032 were downregulated (≥1.5-fold decrease; *P* < 0.05 for all).

**Figure 1:**
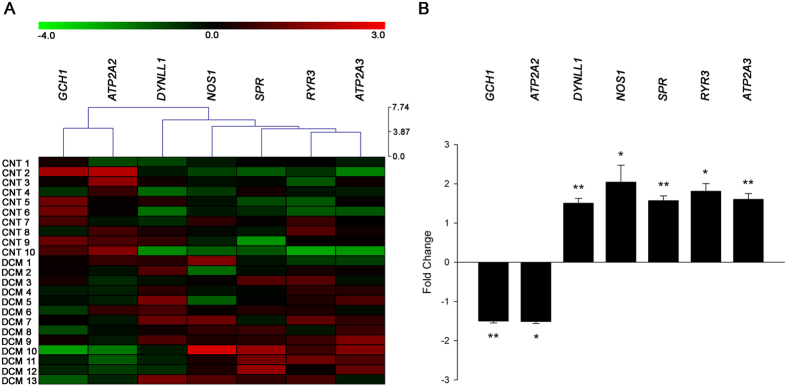
mRNA expression levels of altered NOS1-related genes involved in regulating the physiological function of myocytes in human dilated hearts. Heat map and hierarchical clustering based on the fold change (FC) values. The heat map and hierarchical clustering analyses show the separation of the DCM and CNT groups. Columns: genes; rows: samples. Colours depict the relative expression level of each gene; blue: lowest, yellow: highest (**A**). The graph shows the values obtained by RNA-sequencing. The values from the controls were set to 1. FC units represent the fold induction over the CNT mRNA relative expression levels. Bars indicate FC ± SEM (standard error of the mean) (**B**). *P < 0.05 and **P < 0.01 versus the CNT group.

We focused on 28 NOS1-related genes involved in the regulation of myocardial Ca^2+^ fluxes and found significant differences in seven NOS1-related genes between the DCM samples and the CNTs. A heat map and hierarchical clustering were performed using MeV (v. 4.9.0) program to compare the altered genes in DCM samples with the corresponding genes in CNTs. Notably, this analysis identified two divergent gene expression profiles, showing a clear demarcation between the DCM and the CNT groups (Fig. 1A).

As shown in Table 2 and Fig. 1B, *NOS1* was overexpressed in the DCM samples compared to the CNTs, while we did not find similar changes in *NOS2* and *NOS3*. Important NOS1-related genes such as *GCH1* and *ATP2A2* had significantly decreased mRNA levels in the DCM samples, while mRNA levels of *SPR, DYNLL1, ATP2A3,* and *RyR3* were upregulated. RT-qPCR analysis validated these results. We found that the expression of *NOS1* (3.781 fold, *P* < 0.05), *DYNLL1* (2.854 fold*, P* < 0.05) and *SPR (*3.018 fold, *P* < 0.05) were higher in DCM group than in CNT, and *GCH1* mRNA levels were decreased (−1.320 fold*, P* < 0.05).

### NOS1 protein levels, activity, and protein inhibitor of NOS1 (PIN) in human dilated hearts

**Figure 2:**
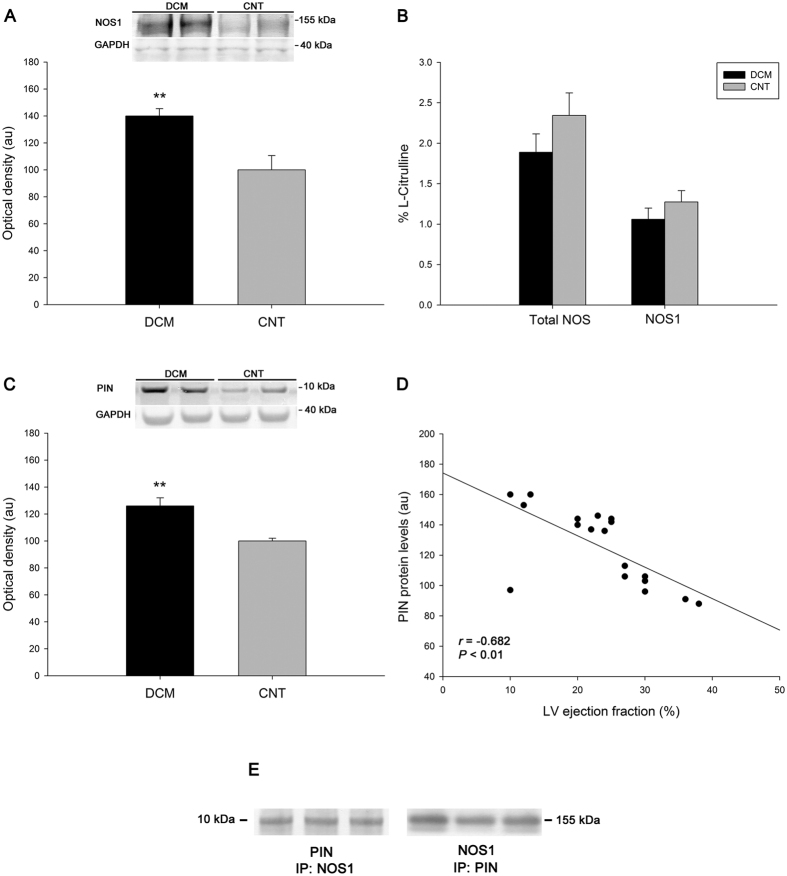
NOS1 protein levels, activity, and protein inhibitor of NOS1 (PIN) in human dilated hearts. NOS1 protein levels (**A**). Total NOS and NOS1 activity (**B**). PIN protein levels (**C**). Relationship between PIN protein levels and left ventricular ejection fraction (**D**). Association between NOS1 and PIN. Immunoprecipitates (IP) were immunoblotted for NOS1 and PIN, respectively. For the protein analysis (**A**,**C**), the values from the CNT group were set to 100. The data are expressed as optical density, arbitrary units (au). The values were normalised to GAPDH and finally to the CNTs. All data are expressed as means ± SEM (standard error of the mean). **P < 0.01 versus the CNT group.

Using western blotting, with total heart samples increased to 30, NOS1 protein levels were significantly increased in the DCM samples, as shown in Fig. 2A (140 ± 24 au versus 100 ± 34 au, *P* < 0.01). However, total NOS and NOS1 activity did not reach statistical significance when we compared pathological and CNT hearts (Fig. 2B). In addition, we found an increase in PIN levels in DCM hearts (126 ± 25 au versus 100 ± 6 au, *P* < 0.01) (Fig. 2C). Interestingly, we observed a significant negative correlation between PIN protein levels and LV ejection fraction (*r* = −0.682, *P* < 0.01, Fig. 2D). The complete parameters of LV function were available for 18 of 20 samples. We found protein-protein interactions of NOS1 with PIN (Fig. 2E) and a positive correlation between the levels of these proteins (*r* = 0.729, *P* < 0.01).

### Changes in GCH1 and SPR protein levels in human dilated hearts and tetrahydrobiopterin biosynthesis

**Figure 3:**
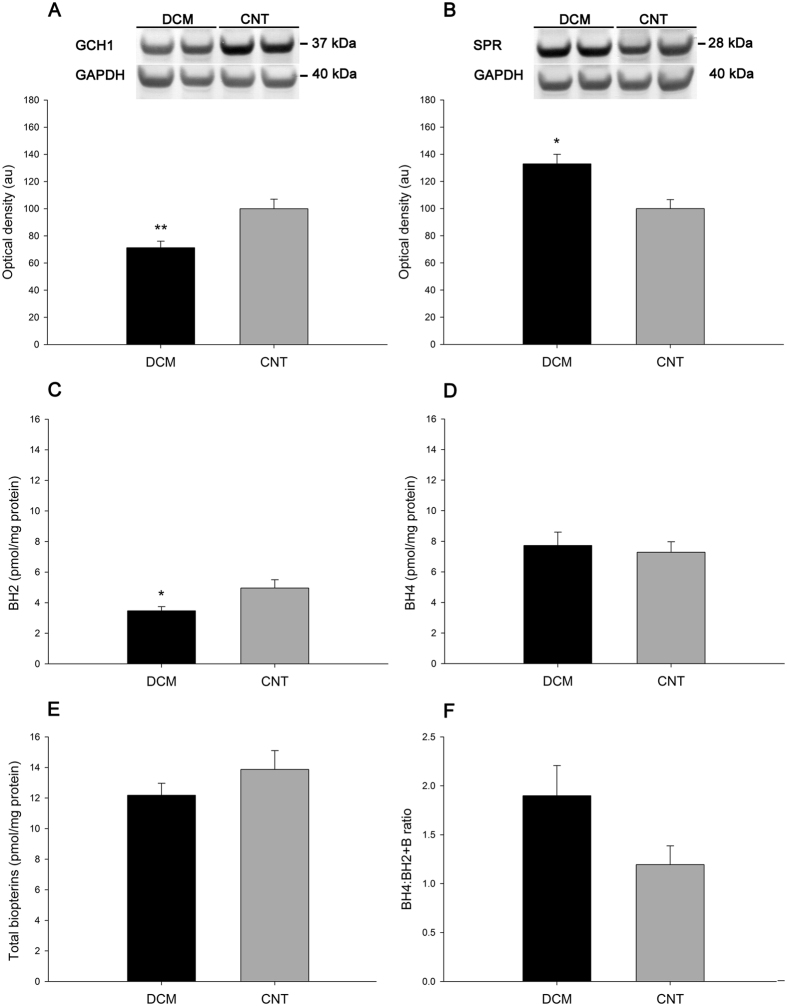
Differential GCH1 and SPR protein levels in human dilated hearts and tetrahydrobiopterin biosynthesis. GCH1 (**A**) and SPR (**B**) protein levels. Values from the CNT group were set to 100. The data are expressed in optical density, arbitrary units (au). The values were normalised to GAPDH and finally to the CNT group. Biopterin content: BH2 (**C**), BH4 (**D**), total biopterins (**E**), BH4:BH2 + B ratio (**F**). All data are expressed as means ± SEM (standard error of the mean). *P < 0.05 and **P < 0.01 versus the CNT group.

The protein levels of GCH1 and SPR also differed between the DCM and CNT groups. The DCM group had lower levels of GCH1 (71 ± 21 au versus 100 ± 22 au, *P* < 0.01; Fig. 3A) and higher levels of SPR (126 ± 23 au versus 100 ± 34 au, *P* < 0.05; Fig. 3B) than the CNT group.

To evaluate whether downregulated myocardial GCH1 leads to a decrease in biopterin content in pathological hearts, we compared the biopterin concentrations of DCM and CNT samples. As shown in Fig. 3C, we detected a small increase in BH2 in CNT hearts (3.47 ± 0.88 pmol/mg protein versus 4.96 ± 1.33 pmol/mg, *P* < 0.05). By contrast, we did not find differences in the BH4 level, total biopterins level, and BH4:BH2 + B ratio (7.73 ± 2.77 pmol/mg protein versus 7.28 ± 1.70 pmol/mg protein, 12.18 ± 2.48 pmol/mg protein versus 13.87 ± 3.02 pmol/mg protein, and 1.90 ± 0.98 versus 1.19 ± 0.47; respectively, Fig. 3D–F).

## Discussion

In the present study we reveal that the upregulation of cardiac NOS1 in human dilated hearts is not accompanied by an increase in NOS activity or by alterations in BH4 levels. BH4 is an important regulator of cardiovascular homeostasis^5^,^7^,^13^. Several studies indicates that maintaining adequate BH4 levels in the endothelium is likely to be critical in regulating the balance between NO and O_2_^−^ synthesis^5^,^8^. To date, studies have largely focused on the role of BH4 in inflammation and vascular pathologies^14^,^15^,^16^,^17^, but less is known about its role in the heart. Thus, the cardiomyocyte-specific implications of BH4 in cardiac pathologies remain unclear. Previous works have demonstrated that pressure overload induced by transverse aortic constriction in mice reduces cardiac BH4 levels, leading to myocyte hypertrophy, cardiac dilation, interstitial fibrosis, and ventricular dysfunction^8^. In addition, the effect of NOS uncoupling in a mouse model of diastolic dysfunction was shown to result in increased cardiac oxidative stress, principally from uncoupled NOS1, and an reduced BH4:oxidised biopterins ratio^18^. A key factor for NO production by NOS1 is the presence of BH4, regulated by GCH1, the rate-limiting enzyme in BH4 biosynthesis, which catalyses the formation of dihydroneopterin 3’triphosfate from GTP, producing BH4 in two further steps through the activities of 6-pyruvoyltetrahydropterin synthase and SPR^5^,^7^,^8^. In the present work, we found an increase in cardiac NOS1 levels in pathological hearts, but in no change in its activity. Thus, we aimed to identify the possible factors, which are responsible for the NOS1 activity to be similar in dilated and control hearts in spite of the elevated levels of NOS1 found in the pathological samples. We measured the biopterin concentrations in the DCM and CNT samples and did not find changes in the BH4 level, total biopterin level, or BH4:BH2 + B ratio, despite finding significantly lower levels of GCH1. This decrease in GCH1 could be compensated for the high levels of SPR found in these dilated hearts, highlighting the role of SPR as a compensatory factor to maintain the normal levels of biopterins in conditions of low GCH1 concentrations and supporting previous results from an experimental model^14^.

Other essential molecules involved in the regulation of NOS1 activity, such as calmodulin, heat shock protein 90, and NOS interacting protein, did not differ between the DCM and CNT groups. However, we found a substantial increase in expression and protein levels of PIN (*DYNLL1* gene) in DCM hearts and a good relationship with NOS1 levels. Although is it not possible to determine if NOS1 activity show dose-dependency in our samples under the assay conditions, these results could help explain the lack of change in NOS1 activity despite the high levels found in the pathological samples, as PIN physically interacts and inhibits the activity of NOS1^11^. Fan *et al.* purified large quantities of PIN overexpressed in bacterial cells and showed that the PIN-binding region of NOS1 specifically mapped to a 17-residue peptide fragment from Met-228 to His-244 of NOS1. They also reported that PIN binds to NOS1 with a 1:2 stoichiometry^19^. We found a significant relationship between PIN protein levels and LV ejection fraction, indicating that increased PIN levels are associated with functional deterioration and supporting the direct involvement of this inhibitor in myocardial Ca^2+^ homeostasis. Therefore, our findings indicate new interesting directions for research on dilated function pathogenesis and may provide targets in this complex process that deserve further investigation.

In addition, we detected the presence of important alterations in different NOS1-related counterparts also involved in the regulation of the physiological function of cardiomyocytes in the cardiac tissue of DCM patients. The central role of the cardiac sarcoplasmic reticulum and its Ca^2+^-handling proteins, including RyRs, SERCA2a, and phospholamban (PLB), in excitation-contraction coupling is well known^20^,^21^,^22^,^23^. NOS1 is normally located in the sarcoplasmic reticulum, where it associates with RyR, and it is involved in the activities of calcium-handling molecules. In HF, the level and activity of SERCA2a are decreased, contributing directly to impaired cardiac contraction and relaxation^20^,^21^. It has been reported that SERCA2a is impaired in the absence of NOS1^24^. As expected, we found *SERCA2a* to be downregulated in our study. Furthermore, we observed alterations in the expression of *RyR3* but not in that of *RyR2*; this result could open an unexplored area of research into the role of this isoform in HF.

A common limitation of studies that examine cardiac tissues from end-stage HF patients is the presence of extensive variability in both disease aetiology and treatment.

In conclusion, we demonstrate that the upregulation of cardiac NOS1 is not accompanied by an increase in NOS1 activity in dilated hearts, due in part to the elevated levels of PIN found and not to alterations in biopterins biosynthesis. Notably, the protein concentrations of PIN were significantly associated with impaired ventricular function, highlighting the importance of this inhibitor of NOS1 activity in Ca^2+^ homeostasis. These results take a central role in the current list of targets for future studies focused on the complex cardiac dysfunction process that occurs in DCM through more efficient harnessing of NOS signalling.

## Methods

### Collection of samples

Experiments were performed with LV samples from 30 explanted human hearts: 20 from patients with DCM undergoing cardiac transplantation and 10 from non-diseased CNTs. Clinical history, hemodynamic study, ECG, and Doppler echocardiography data were available from these patients. Non-ischemic DCM was diagnosed when patients had LV systolic dysfunction (EF < 40%) with a dilated non-hypertrophic left ventricle (LVEDD > 55 mm) on echocardiography. Moreover, patients did not show existence of primary valvular disease and familial DCM. All patients were functionally classified according to the New York Heart Association (NYHA) criteria and they were receiving medical treatment following the guidelines of the European Society of Cardiology^25^. Clinical characteristics of patients are summarised in Table 1.

All CNTs had normal LV function (EF > 50%), as determined by Doppler echocardiography. None had any history of cardiac disease. CNT samples were obtained from non-diseased donor hearts that had been rejected for cardiac transplantation owing to size or blood type incompatibility, or due to non-availability of a suitable recipient within the requisite time period. All these donors died of either cerebrovascular or motor vehicle accidents.

Our hospital has been ranked as the national leader in heart transplantation for the third time with more than 700 transplants accomplished in the last 25 years. In accordance we acquire high-quality samples as revealed by high values (greater or equal to 9) of RNA integrity number (RIN). We had access to operating rooms during interventions and full explanted hearts in all cases. For each procedure, we chose tissue samples from the same area of the left ventricle to standardize our research methodology. In our study, all tissue samples were obtained from near the apex of the left ventricle, were maintained in 0.9% NaCl, and were preserved at 4 °C for a maximum of 6 hours after the loss of coronary circulation. Samples were stored at −80 °C until used.

This study was approved by the Ethics Committee (Biomedical Investigation Ethics Committee of La Fe University Hospital of Valencia, Spain). Prior to tissue collection, signed informed consent was obtained from each patient. The study was conducted in accordance with the guidelines of the Declaration of Helsinki^26^.

### RNA sequencing, RT-qPCR validation, and protein analysis

Methods used for RNA extraction, RNA-seq and computational analysis of the RNA-seq data were performed as previously described^27^. For this analysis, LV tissue samples were obtained from 13 DCM patients and 10 non-diseased CNTs. The data presented in this publication have been deposited in the NCBI Gene Expression Omnibus (GEO) and can be retrieved using the GEO Series accession number GSE55296 (http://www.ncbi.nlm.nih.gov/geo/query/acc.cgi?acc=GSE55296). Methods used for RT-qPCR analysis are included as Supplementary Information and to improve the numerical base with a higher number of samples in this validation we increased the DCM samples up to 20. We determined the protein levels of NOS1, PIN, GCH1, and SPR with total heart samples increased to 30. We also studied protein-protein interactions of NOS1 with PIN. Data are included as Supplementary Information.

### Nitric oxide synthase 1 activity and tetrahydrobiopterins determination

Total NOS and NOS1 activity and myocardial biopterins were assessed by high performance liquid chromatography (HPLC), as described previously^7^,^28^. Details of these methods are included in the Supplementary Information.

### Statistical methods

Data were expressed as mean ± standard deviation for continuous variables and as percentage for discrete variables. Significant mean differences between groups were analysed using the Mann-Whitney U test. Spearman’s correlation coefficients were calculated to determine the relationships between altered proteins and echocardiographic parameters. *P* <* *0.05 was considered statistically significant. All statistical analysis was performed using the SPSS software for Windows (version 20.0; IBM SPSS Inc; Chicago. IL, USA).

## Additional Information

**How to cite this article**: Roselló-Lletí, E. *et al.* Protein Inhibitor of NOS1 Plays a Central Role in the Regulation of NOS1 Activity in Human Dilated Hearts. *Sci. Rep.* **6**, 30902; doi: 10.1038/srep30902 (2016).

## Supplementary Material

Supplementary Information

## Figures and Tables

**Table 1:** Clinical characteristics of patients with dilated cardiomyopathy.

	RNA-seq analysis	Protein analysis RT-qPCR analysis
	DCM (*n* = 13)	DCM (*n* = 20)
Age (years)	51 ± 11	48 ± 13
Gender male (%)	92	82
NYHA class	3.4 ± 0.4	3.3 ± 0.3
Prior smoking (%)	50	56
BMI (kg/m^2^)	27 ± 5	28 ± 6
Total cholesterol (mg/dL)	147 ± 37	139 ± 44
Prior hypertension (%)	17	21
Prior diabetes mellitus (%)	17	18
Hemoglobin (mg/mL)	13 ± 3	13 ± 3
Hematocrit (%)	39 ± 8	39 ± 7
Duration of disease (months)	75 ± 68	68 ± 63
Echo-Doppler study		
EF (%)	20 ± 7	22 ± 9
FS (%)	11 ± 4	12 ± 5
LVESD (mm)	71 ± 12	67 ± 12
LVEDD (mm)	80 ± 11	76 ± 11
LVMI (g/m^2^)	241 ± 77	230 ± 75

Data are showed as the mean value ± SD. DCM, dilated cardiomyopathy; NYHA, New York Heart Association; BMI, body mass index; EF, ejection fraction; FS, fractional shortening; LVESD, left ventricular end-systolic diameter; LVEDD, left ventricular end-diastolic diameter; LVMI, left ventricular mass index.

**Table 2:** Nitric oxide synthases (NOS) and NOS1- related molecules in human dilated cardiomyopathy.

Gene name	Description	FC ± SD	p value
*NOS1*	nitric oxide synthase 1	2.04 ± 1.55	*P* < 0.05
*NOS2*	nitric oxide synthase 2	−1.23 ± 0.80	NS
*NOS3*	nitric oxide synthase 3	−1.05 ± 0.36	NS
*GCH1*	GTP cyclohydrolase 1	−1.50 ± 0.18	*P* < 0.01
*XDH*	xanthine dehydrogenase	−1.39 ± 0.58	NS
*RYR1*	ryanodine receptor 1	1.37 ± 1.10	NS
*RYR2*	ryanodine receptor 2	−1.17 ± 0.21	NS
*RYR3*	ryanodine receptor 3	1.81 ± 0.71	*P* < 0.05
*PLN*	phospholamban	−1.24 ± 0.28	NS
*ATP2A1*	ATPase, Ca^2+^ transporting, cardiac muscle, fast twitch (SERCA1)	−1.24 ± 0.61	NS
*ATP2A2*	ATPase, Ca^2+^ transporting, cardiac muscle, slow twitch 2 (SERCA2)	−1.51 ± 0.19	*P* < 0.05
*ATP2A3*	ATPase, Ca^2+^ transporting, ubiquitous (SERCA3)	1.60 ± 0.53	*P* < 0.01
*PRKG1*	protein kinase, cGMP-dependent, type I	−1.08 ± 0.19	NS
*PRKG2*	protein kinase, cGMP-dependent, type II	1.44 ± 2.25	NS
*SPR*	sepiapterin reductase	1.57 ± 0.45	*P* < 0.01
*NOS1AP*	nitric oxide synthase 1 adaptor protein	1.09 ± 0.53	NS
*CALM1*	calmodulin 1	−1.03 ± 0.10	NS
*CALM2*	calmodulin 2	1.00 ± 0.20	NS
*CALM3*	calmodulin 3	1.23 ± 0.23	NS
*CAV1*	caveolin 1	−1.17 ± 0.26	NS
*CAV3*	caveolin 3	1.18 ± 0.29	NS
*HSP90AA1*	heat shock protein 90 kDa alpha (cytosolic), class A member 1	−1.49 ± 0.11	NS
*HSP90AB1*	heat shock protein 90 kDa alpha (cytosolic), class B member 1	−1.06 ± 0.25	NS
*DYNLL1*	dynein, light chain, LC8-type 1	1.51 ± 0.44	*P* < 0.01
*NOSIP*	nitric oxide synthase interacting protein	1.21 ± 0.31	NS
*PFKM*	phosphofructokinase, muscle	−1.23 ± 0.32	NS
*SNTA1*	syntrophin, alpha 1	1.03 ± 0.26	NS
*DMD*	dystrophin	1.01 ± 0.19	NS

Data are showed as the fold change (FC) value ± standard deviation (SD).
