# Supplementary material for: Protein Inhibitor of NOS1 Plays a Central Role in the Regulation of NOS1 Activity in Human Dilated Hearts
Source: Sci Rep. 2016 Aug 2;6:30902. doi: 10.1038/srep30902 (PMC4969592; doi:10.1038/srep30902)
Supplement: Supplementary Information [file srep30902-s1.pdf]

PROTEIN INHIBITOR OF NOS1 PLAYS A CENTRAL ROLE IN THE REGULATION OF NOS1 ACTIVITY IN HUMAN DILATED HEARTS

Esther Roselló-Lletí[1†*], PhD; Estefanía Tarazón[1†], PhD; Ana Ortega[1], PhD; Carolina Gil-Cayuela[1], PhD; Ricardo Carnicer[2], PhD; Francisca Lago[3], PhD; Jose Ramón González-Juanatey[3], MD, PhD; Manuel Portolés[1], PhD; Miguel Rivera[1], MD, PhD, FACC

[1]Cardiocirculatory Unit, Health Research Institute Hospital La Fe (IIS La Fe), Valencia, Spain; [2]Department of Cardiovascular Medicine, University of Oxford, United Kingdom.[3]Cellular and Molecular Cardiology Research Unit, Department of Cardiology and Institute of Biomedical Research, University Clinical Hospital, Santiago de Compostela, Spain. †The first two authors contributed equally to the study.

SUPPLEMENTARY INFORMATION

SUPPLEMETARY METHODS

RNA extraction

Heart samples were homogenised in TRIzol® reagent in a TissueLysser LT (Qiagen, UK). All RNA extractions were performed using a PureLink™ Kit according to the manufacturer's instructions (Ambion Life Technologies, CA, USA). RNA was quantified using a NanoDrop1000 spectrophotometer (Thermo Fisher Scientific, UK), and the purity and integrity of the RNA samples were measured using an Agilent 2100 Bioanalyzer with an RNA 6000 Nano LabChip kit (Agilent Technologies, Spain). All samples showed a 260/280 ratio >2.0 and an RNA integrity number ≥9.

RNA-seq

The RNA samples were isolated using a MicroPoly(A) Purist Kit™ (Ambion, USA). The total polyA-RNA samples were used to generate whole transcriptome libraries that were sequenced on a SOLiD 5500XL platform as per the manufacturer's recommendations (Life Technologies, CA). The amplified cDNA quality was analysed using the Bioanalyzer 2100 DNA 1000 kit (Agilent Technologies, Spain), and the cDNA was quantified using the Qubit 2.0 Fluorometer (Invitrogen, UK). Whole transcriptome libraries were used to generate SOLiD templated beads by following the SOLiD Templated Bead Preparation guide. Bead quality was estimated based on WFA (workflow analysis) parameters. The samples were sequenced using the 50625 paired-end protocol, which generated 75 nt + 35 nt (Paired-End) + 5 nt (Barcode) sequences. Quality data were measured using the SETS software parameters (SOLiD Experimental Tracking System).

Computational analysis of RNA-seq data

The initial whole transcriptome paired-end reads obtained from the sequencing were mapped against the latest version of the human genome (Version GRchr37/hg19) by using the Life Technologies mapping algorithm (http://www.lifetechnologies.com/). The aligned records were reported in the BAM/SAM format[1]. Bad quality reads (Phred score <10) were eliminated using the Picard Tools software[2]. The isoform and gene predictions were subsequently estimated using the cufflinks method[3], and the expression levels were calculated using the HTSeq software[4]. The Edge method was applied to analyse the differential expression between conditions[5]. This method relies on a Poisson model to estimate the RNA-seq data variance for differential expression. We selected genes and isoforms that were calculated to exhibit $P<0.05$ and fold-change >1.5.

RT-qPCR analysis

Reverse transcription was carried out using 1 μg total RNA and M-MLV enzyme (Invitrogen Ltd, UK) according to the manufacturer's protocol. The resulting cDNA was used as a template for RT-qPCR in a high throughput thermocycler (ViiA™ 7 Real-Time RT-PCR System, Applied Biosystems, Foster City, CA, USA) according to the manufacturer's instructions using TaqMan® probes for *NOS1* (Hs00167223_m1), *DYNLL1* (Hs00853309_g1), *SRP* (Hs00268403_m1) and *GCH1* (Hs00609198_m1). Quantification of gene expression was normalized to *GAPDH* (Hs99999905_m1), *PGK1* (Hs99999906_m1), and *TFRC* (Hs00951083_m1) as endogenous controls. Relative gene expression levels were calculated using the $2^{-\Delta\Delta CT}$ method[6].

Protein analysis

Twenty-five milligrams of frozen left ventricle was transferred into Lysing Matrix D tubes designed for use with the FastPrep-24 homogeniser (MP Biomedicals, USA) in a total protein extraction buffer (2% SDS, 10 mM EDTA, 6 mM Tris–HCl, pH 7.4) with protease inhibitors (25 µg/mL aprotinin and 10 µg/mL leupeptin). The homogenates were centrifuged at 13200 rpm and the supernatant was aliquoted. The protein content of the aliquot was determined using Peterson's modification of the micro Lowry method with bovine serum albumin (BSA) as the standard.

We determined the protein levels with total heart samples increased to 30. Protein samples for detecting NOS1 were separated using Tris-Acetate Midi gel electrophoresis with 3–8% polyacrylamide, and GCH1, SRP, and PIN were separated using Bis-Tris electrophoresis on 4–12% polyacrylamide gels under reducing conditions. After electrophoresis, the proteins were transferred from the gel to a PVDF membrane using the iBlot Dry Blotting System (Invitrogen Ltd, UK) for western blot analysis. The membranes were blocked overnight at 4°C with 1% BSA in Tris buffer solution containing 0.05% Tween 20 and thereafter, were incubated for 2 hours with the primary antibody in the same buffer. The primary detection antibodies used were anti-NOS1 mouse monoclonal antibody (1:100, obtained from Santa Cruz Biotechnology, INC), anti-GCH1 rabbit polyclonal (1:1000, obtained from Abcam, Cambridge, UK), anti-SRP rabbit monoclonal antibody (1:1000, obtained from Abcam, Cambridge, UK), anti-DYNLL1 rabbit monoclonal antibody (1:6000, obtained from Abcam, Cambridge, UK), and anti-GAPDH mouse monoclonal antibody (1:2000, obtained from Abcam, Cambridge, UK) as a loading control.

The bands were visualised using an acid phosphatase-conjugated secondary antibody and nitro blue tetrazolium/5-bromo-4-chloro-3-indolyl phosphate (NBT/BCIP, Sigma-Aldrich, St. Louis, USA) substrate system. Finally, the bands were digitalized using an image analyzer

(DNR Bio-Imagining Systems, Israel) and quantified with the GelQuant Pro (v. 12.2) program.

Immunoprecipitation

For immnunoprecipitation, lysate proteins of human hearts were incubated overnight at 4 °C under gentle rotation with either DYNLL1 rabbit monoclonal antibody (obtained from from Abcam, Cambridge, UK), or NOS1 monoclonal antibody (obtained from Santa Cruz Biotechnology, INC) at a final concentration of 5 μg/mL in 100 μL of lysis buffer. High affinity Protein A or G-conjugated agarose (obtained from Abcam, Cambridge, UK; 20 μL) were added for 3 hours at 4 °C, centrifugated, and washed three times. This was followed by resuspending the pellet with 20 μL SDS sample buffer and heating the samples to 95 °C for 5 minutes. Thereafter, samples were separated by electrophoresis and immunoblotted.

NOS activity

NOS activity was measured using radiochemical detection of L-arginine to L-citrulline conversion, as described previously.[7] Briefly, separation of the products of L-arginine metabolism was obtained by ion exchange chromatography (Jasco Ltd.) and on-line radiochemical scintillation detection (Lablogic Systems Ltd). Recorded data were analysed using Azur software (Datalys, France). LV was homogenized ice-cold Krebs' HEPES Buffer containing 5 μmol/L or-NOHA (to inhibit arginase activity). After centrifugation (13,000 rpm for 10 mins at 4°C), the supernatant was then incubated for 30 mins on ice with added NOS cofactors except BH4 (i.e., 10 μmol/L FAD, 10 μmol/L FMN, 1 mmol/L NADPH), in the presence or absence of either the non-specific NOS inhibitor, L-NAME (1 mmol/L), or the NOS1-selective inhibitor SMTC (100 nmol/L), followed by 4 hours incubation at 37°C with 3

μL of labelled ^{14}C L-arginine (Amersham Biosciences UK Ltd.). Trichloroacetic acid (10%) was then added to de-proteinate the samples, prior to centrifugation. The supernatant was placed into the auto-sampler cooled to 4ºC for chromatographic analysis. Standards of ^{14}C-labelled Larginine (1 μmol/L), L-citrulline (0.1 μmol/L), and L-ornithine (0.2 μmol/L, all from Amersham Bioscience UK Ltd.) were used to determine elution time. Chromatographic peaks were integrated and expressed as a proportion of total ^{14}C counts for each sample. Results were expressed as the L-NAME- or SMTC-inhibitable fraction. NOS activity was normalised (500 μg of protein per reaction).

Biopterins determinations

The detection of the different biopterins BH4, BH2 and B was performed by separation and quantification by reverse phase HPLC.[8] Tissues were prepared from frozen (in -80°C storage); 20-30 mg of LV samples were homogenised as previously described in 500 μl ice cold re-suspension buffer, followed by 15 min centrifugation at 13000 rpm and 4ºC. A volume of 180 μl of the supernatant was taken from samples and standards and added to Eppendorf tubes containing 20 μl of ice-cold 10x acid precipitation buffer. These were mixed well by vortexing and centrifuged for 5 min at 13000 rpm (4ºC). Supernatants were injected into the isocratic HPLC system; biopterins were separated using a Carbon-18 column (Hichrom) with a flow of mobile phase at the rate of 1.3 ml/min. Biopterins were quantified by sequential electrochemical (Coulochem III, ESA Inc, USA) and fluorescence (Jasco Ltd) detection. Biopterin concentrations were quantified by comparison with BH4, BH2 and B reference standards and normalised to protein content.

SUPPLEMENTAL REFERENCES

1. Li, H. The Sequence Alignment/Map format and SAMtools. *Bioinformatics.* **25**, 2078-2079 (2009).

2. McKenna, A., et al. The Genome Analysis Toolkit: a MapReduce framework for analyzing next-generation DNA sequencing data. *Genome Res.* **20**, 1297-1303 (2010).

3. Trapnell, C., et al. Transcript assembly and quantification by RNASeq reveals unannotated transcripts and isoform switching during cell differentiation. *Nat Biotechnol.* 28, 511-515 (2010).

4. Anders, S. & Huber, W. Differential expression analysis for sequence count data. *Genome Biol.* **11**, R106 (2010).

5. Robinson, M.D., McCarthy, D.J., & Smyth, G.K. EdgeR: a Bioconductor package for differential expression analysis of digital gene expression data. *Bioinformatics.* **26**, 139-140 (2010).

6. Livak, K.J., Schmittgen, T.D. Analysis of relative gene expression data using real-time quantitative PCR and the 2(-Delta Delta C(T)) method. *Methods*. **25**: 402-408 (2001).

7. de Bono, J.P., Warrick, N., Bendall, J.K., Channon, K.M. & Alp, N.J. Radiochemical HPLC detection of arginine metabolism: measurement of nitric oxide synthesis and arginase activity in vascular tissue. Nitric Oxide. **16**, 1-9 (2007).

8. Crabtree, M. J., et al. Quantitative regulation of intracellular endothelial nitric-oxide synthase (eNOS) coupling by both tetrahydrobiopterin-eNOS stoichiometry and biopterin redox status: insights from cells with tet-regulated GTP cyclohydrolase I expression. *J Biol Chem*. **284**, 1136-1144 (2009).
